# Histone modification of pain-related gene expression in spinal cord neurons under a persistent postsurgical pain-like state by electrocautery

**DOI:** 10.1186/s13041-021-00854-y

**Published:** 2021-09-20

**Authors:** Yosuke Katsuda, Kenichi Tanaka, Tomohisa Mori, Michiko Narita, Hideyuki Takeshima, Takashige Kondo, Yoshiyuki Yamabe, Misa Matsufuji, Daisuke Sato, Yusuke Hamada, Keisuke Yamaguchi, Toshikazu Ushijima, Eiichi Inada, Naoko Kuzumaki, Masako Iseki, Minoru Narita

**Affiliations:** 1 Department of Anesthesiology and Pain Medicine, Juntendo University Graduate School of Medicine, 2-1-1 Hongo, Bunkyo-ku, Tokyo, 113-8421 Japan; 2 Department of Pharmacology, Hoshi University School of Pharmacy and Pharmaceutical Sciences, 2-4-41 Ebara, Shinagawa-ku, Tokyo, 142-8501 Japan; 3 Division of Cancer Pathophysiology, National Cancer Center Research Institute, 5-1-1 Tsukiji, Chuo-ku, Tokyo, 104-0045 Japan; 4 Department of Molecular and Cellular Medicine, Institute of Medical Science, Tokyo Medical University, 6-7-1 Nishishinjuku, Shinjuku-ku, Tokyo, 160-0023 Japan; 5 Division of Epigenomics, National Cancer Center Research Institute, 5-1-1 Tsukiji, Chuo-ku, Tokyo, 104-0045 Japan; 6 Department of Anesthesiology and Pain Medicine, Juntendo Tokyo Koto Geriatric Medical Center, 3-3-20 Shinsuna, Koto-ku, Tokyo, 136-0075 Japan

**Keywords:** Chronic postsurgical pain, Spinal cord, Synaptic plasticity, Epigenetics

## Abstract

Chronic postsurgical pain (CPSP) is a serious problem. We developed a mouse model of CPSP induced by electrocautery and examined the mechanism of CPSP. In this mouse model, while both incision and electrocautery each produced acute allodynia, persistent allodynia was only observed after electrocautery. Under these conditions, we found that the mRNA levels of Small proline rich protein 1A (Sprr1a) and Annexin A10 (Anxa10), which are the key modulators of neuropathic pain, in the spinal cord were more potently and persistently increased by electrocautery than by incision. Furthermore, these genes were overexpressed almost exclusively in chronic postsurgical pain-activated neurons. This event was associated with decreased levels of tri-methylated histone H3 at Lys27 and increased levels of acetylated histone H3 at Lys27 at their promoter regions. On the other hand, persistent allodynia and overexpression of Sprr1a and Anxa10 after electrocautery were dramatically suppressed by systemic administration of GSK-J4, which is a selective H3K27 demethylase inhibitor. These results suggest that the effects of electrocautery contribute to CPSP along with synaptic plasticity and epigenetic modification.

**Supplementary Information:**

The online version contains supplementary material available at 10.1186/s13041-021-00854-y.

## Introduction

Pain, which is a common medical problem, is an unpleasant sensation caused by illness or injury. Although acute pain itself alerts us to the presence of noxious stimuli, persistent pain does not provide a similar warning function. Chronic pain is defined as pain that lasts longer than several months, and can be caused by various factors (e.g., tissue injuries, aging accompanied by joint and bone damage, nerve injuries and surgical incisions) [1]. Such ongoing pain, which is resistant to medical treatment, reduces the patient’s quality of life (QOL) and could be a risk factor for depression [2, 3]. Thus, preventing the development of chronic pain is an important tactic for improving the QOL of patients.

In most cases, acute postsurgical pain, which is a form of nociceptive pain that is temporarily observed after surgery, can be controlled by analgesic medications and disappears with healing. On the other hand, chronic postsurgical pain (CPSP) is believed to be associated with nerve injury during surgery [4]. However, the mechanism that underlies the establishment of CPSP after surgery remains unclear, and options for the treatment of CPSP are far from satisfactory.

Electrocautery is a well-known routine surgical procedure that enables faster surgeries, achieves better hemostasis, removes abnormal tissue growth and prevents infection. However, it also generates heat and produces tissue and neuronal damage, which may result in CPSP [5, 6]. Therefore, there may be intrinsic differences between traditional incision and electrocautery with respect to the induction of CPSP. While some studies have compared postsurgical pain resulting from incision by scalpel to that caused by electrocautery, they focused on acute postsurgical pain.

To better understand the mechanism of persistent pain, several animal models have been developed. Particularly, Brennan et al. [7] established a model of postsurgical pain using rats that involved incision into the plantar surface of the hind paw, which induces allodynia in response to mechanical stimuli. However, there is no animal model for studying the progress of pain after electrocautery. In the present study, we compared the relative maintenance of allodynia after incision according to Brennan’s model [7] and electrocautery in the hind paw of mice.

Recently, epigenetic modifications have been reported to contribute to prolongation of pathophysiological pain. In our previous study, we found that increased expression of chemokine (C–C motif) ligand 7 (Ccl7, also known as monocyte chemotactic protein 3) in spinal astrocytes associated with decreased trimethylation of histone H3 at Lys27 (H3K27me3) at the Ccl7 promoter was induced by nerve injury [8]. Furthermore, such epigenetic changes promoted pain sensation via enhanced interaction between astrocytes and microglia in the spinal dorsal horn [8]. These findings suggest that histone modifications may also play a role in the prolongation of postsurgical pain. Therefore, we investigated possible epigenetic modifications associated with persistent pain in spinal cord cells induced by electrocautery.

## Methods

### Animals

Male C57BL/6 J mice (7–10 weeks old) (Jackson Laboratory, Bar Harbor, ME, USA) and female cFos-EGFP mice (6–23 weeks old) were used. Female cFos-EGFP mice were obtained by breeding cFos-2AiCreER^T2^ (C57BL/6-Fos < *tm1(icreERT2)Phsh* >) mice (Cyagen Biosciences Inc., Santa Clara, CA, USA) with LSL-EGFP/Rpl10a (B6;129S4-Gt(ROSA)26Sor < *tm9(EGFP/Rpl10a)Amc* > /J) mice (Stock No. 024750, Jackson Laboratory) [9]. Mice had access to food and water ad libitum in a temperature- and humidity-controlled room (24 ± 1 °C, 55 ± 5%, relative humidity) under a 12-h light–dark cycle (light on at 8 a.m.). The behavioral tests were performed during the light phase.

### Generation of Fos-2A-iCreER^T2^ knock-in mice

C57BL/6-Fos < *tm1(icreERT2)Phsh* > (cFos-2AiCreER^T2^) mice, which were based on a C57BL/6 genetic background, were generated at Cyagen Biosciences. The mouse *Fos* gene (NCBI Reference Sequence: NM_010234.2) is located on mouse chromosome 12. Four exons have been identified, with the ATG start codon in exon 1 and the TGA stop codon in exon 4 (Transcript: Fos-201 ENSMUST00000021674.6). To create the P2A-iCreER^T2^ knock-in at the mouse *Fos* locus in C57BL/6 mice, a mixture of Cas9 mRNA, sgRNA, and each targeting vector was injected into fertilized mouse eggs, which were then transferred to surrogate mothers to obtain founder knock-in mice on the B6 background; the TGA stop codon was replaced with the P2A-iCreER^T2^ cassette by CRISPR/Cas-mediated genome engineering.

### Drug administration

Mice received either vehicle or GSK-J4 HCl (2.0 mg/kg, *i.p.*; Selleck, Houston, TX, USA) 1 h before plantar incision and 4 times per day starting the day after surgery. GSK-J4 was prepared daily, in saline (0.9% NaCl) containing 1% DMSO (FUJIFILM Wako Pure Chemical Co. LTD, Osaka, Japan), and mixed at room temperature for at least 1 h before use. Saline containing 1% DMSO used to prepare GSK-J4 was used as a vehicle.

### Plantar incision

**Fig. 1:**
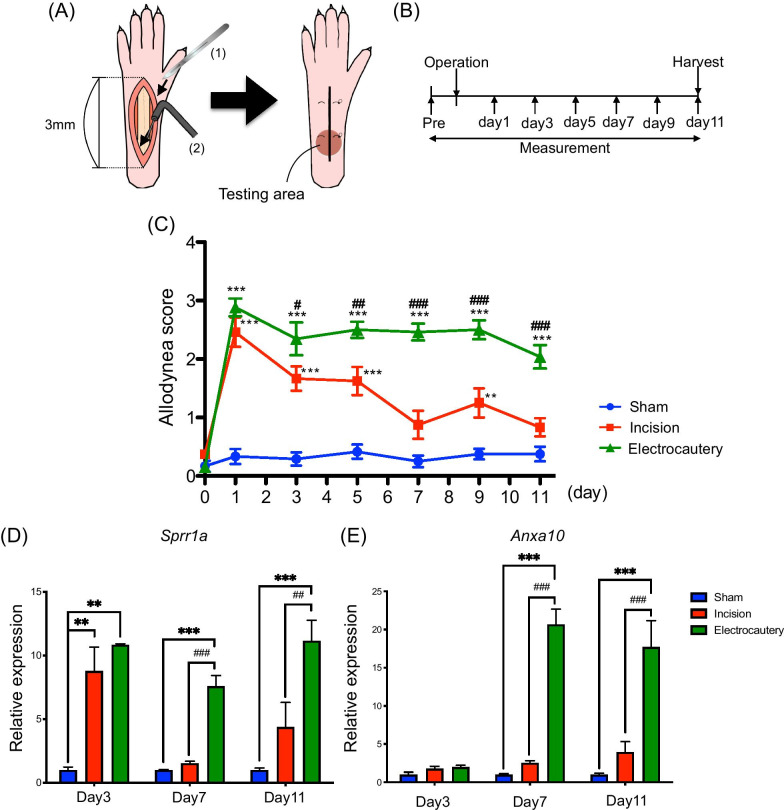
Effect of electrocautery treatment on incision-induced mechanical allodynia in mice. **A** Schematic diagram of the electrocautery operation. (1) A 3 mm longitudinal incision of the skin and fascia of the plantar aspect of the right hind paw of mice was conducted with a number 23 scalpel blade. (2) A 3 mm longitudinal incision of the plantaris muscle was made using a monopolar electrosurgery unit. Finally, the skin was stitched with two mattress sutures of 7–0 nylon. The red circle shows where the von Frey filament was applied (Testing area). **B** Protocol for measurement of the pain threshold. **C** Changes in the pain threshold as measured by the von Frey test (0.16 g). Each point represents the mean ± S.E.M. of 12–13 mice (Two-way repeated measures ANOVA with post-hoc Bonferroni test, **p < 0.01, ***p < 0.001 vs. Sham group. ^#^p < 0.05, ^##^p < 0.01, ^###^p < 0.001 vs. incision group). **D**, **E** mRNA levels of Sprr1a **D** or Anxa10 **E** in the ipsilateral side of the spinal cord were measured at 3, 7 or 11 days after incision or electrocautery. Each column represents the mean ± S.E.M. of 3–9 independent experiments (One-way ANOVA with post-hoc Bonferroni test, **p < 0.01, ***p < 0.001 vs. Sham group, ^##^p < 0.01, ^###^p < 0.001 vs. incision group)

Under isoflurane anesthesia (3% inhalation; FUJIFILM Wako Pure Chemical Co. LTD), a 3-mm longitudinal incision of the skin and fascia of the plantar aspect of the hind paw of mice was conducted with a number 23 scalpel blade, starting 3 mm from the proximal edge of the heel and extending toward the toes. To reproduce Brennan’s model [7] in the mouse, a 3-mm longitudinal incision of the plantaris muscle was made with the scalpel blade, and the skin was then stitched with two mattress sutures of 7–0 nylon. For electrosurgery, longitudinal incision of the skin and fascia of the plantar aspect of the hind paw of mice was performed in a similar manner, and then a 3-mm longitudinal incision of the plantaris muscle was made using a monopolar electrosurgery unit (at 50 W: Vetroson® V-10; Summit Hill Laboratories, Tinton Falls, NJ, USA) with a dispersive electrode pad placed under the body of the mouse. Electrosurgery was conducted while maintaining coagulation and hemostasis of the incision during dissection. The skin was stitched with two mattress sutures of 7–0 nylon (Fig. 1A). In sham-operated mice, the plantaris muscle was exposed without the incision. After surgery, the animals were allowed to recover in their home cages.

### von Frey test for mechanical allodynia

Mechanical allodynia was assessed by the von Frey monofilament test. Briefly, von Frey filaments (0.16 g) were used to poke the mouse hind paw for a maximum of 3 s, and this assessment was conducted three times at 5 s intervals. The withdrawal response of each hind paw after a tactile stimulus was evaluated by scoring as follows: 0, no response; 1, a slow and slight withdrawal response without prolonged flexion (slight lifting of the paw once within 3 s after the stimulus); 2, a slow and prolonged flexion withdrawal response (lifting of the paw within 3 s after the stimulus with sustained lifting during the stimulus); 3, a quick withdrawal response (lifting of the paw immediately after the stimulus with sustained lifting during the stimulus) without flinching or licking; 4, a quick withdrawal response (lifting of the paw immediately after the stimulus) with brisk flinching and/or licking. These withdrawal behaviors were measured twice, and the two scores were averaged. Paw movements associated with locomotion or weight-shifting were omitted from the results. To allow mice to become accustomed to their environment, they were habituated in an acrylic cylinder (15 cm height and 8 cm diameter) on an elevated mesh floor for 1 h before assessment.

### Quantitative reverse transcription polymerase chain reaction (RT-qPCR)

For RT-qPCR analysis, total RNAs were isolated using the mirVana™ miRNA Isolation Kit (Thermo Fisher Scientific Inc., Waltham, MA, USA) from the ipsilateral side of the mouse spinal cord and then first-strand cDNAs were synthesized using the SuperScript® VILO™ cDNA Synthesis Kit (Thermo Fisher). RT-qPCR was conducted using primer pairs and Fast SYBR® Green Master Mix (Thermo Fisher). Glyceraldehyde 3-phosphate dehydrogenase (Gapdh) was used as an internal control for quantification of each sample. Additional file 1: Table S1 contains a complete list of all primers used in this study.

### Labeling and isolation of pain-activated neurons

Eleven days after electrocautery and sham operation in the hind paw of cFos-EGFP mice, these mice were injected with 4-hydroxytamoxifen (4-OHT; 50 mg/kg, *i.p.*). Two hours after 4-OHT injection, mechanical stimulation was applied to the hind paw by a plantar electronic von Frey Anesthesiometer (ALMEMO® 2450 Ahlborn; IITC, Woodland Hills, CA, USA). The pressure of mechanical stimulation by an electronic von Frey Anesthesiometer was increased until the mouse withdrew its hind paw (cut-off pressure: 3.5 g). Seven days after 4-OHT injection, the ipsilateral side of the lumbar spinal cord was collected from cFos-EGFP mice and separated into single cells; debris was removed using an Adult Brain Dissociation kit (Miltenyi Biotec, Bergisch Gladbach, Germany). Hematopoietic cells and their committed precursors were then depleted from single-cell suspension using a Lineage cell depletion kit (Miltenyi Biotec) with magnetic-activated cell sorting (MACS) (autoMACS Pro Separator; Miltenyi Biotec). Neural cells were isolated using a Neuron Isolation kit (Miltenyi Biotec) with MACS and these fractions including neuron-like cells were stained with anti-mouse CD90.2 (Thy1.2)-APC antibody (1:200, BioLegend Inc., San Diego, CA, USA). Subsequently, Thy1.2-APC and cFos-EGFP-positive cells were finally sorted by fluorescence-activated cell sorting (FACS) (FACS Aria III; BD Biosciences, San Jose, CA, USA). Total RNA derived from these sorted neurons was extracted using a PicoPure RNA Isolation Kit (Thermo Fisher) according to the protocol for RNA extraction, and then RT-qPCR was performed after specific transcripts were pre-amplified for 18 cycles using PreAmp Master Mix (Fluidigm Co., South San Francisco, CA, USA).

### Chromatin immunoprecipitation (ChIP)

We performed a chromatin immunoprecipitation assay according to previous studies [8, 10, 11] with minor modifications. Thirty μg of chromatin extracted from the mouse lumbar spinal cord, which was sonicated with a Bioruptor (Sonicbio Co., Ltd., Kanagawa, Japan), was incubated with specific antibodies against acetylated histone H3 at Lys27 (H3K27ac; Abcam, Cambridge, UK) or tri-methylated histone H3 at Lys27 (H3K27me3; Cell Signaling Technology Inc., Danvers, MA, USA) overnight at 4 °C. The immunocomplex was collected with the use of Dynabeads® Protein A (Invitrogen, Carlsbad, CA, USA), and DNA was recovered by treatment with RNaseA and proteinase K refined by phenol/chloroform extraction and isopropanol precipitation. Quantitative PCR was performed as described previously [8, 12]. The detailed primer sequences for Sprr1a and Anxa10 were as follows: Sprr1a forward, 5′-CACCTGGGTTCTCTGTCACC-3′, and reverse, 5′-CAGGACCACTTCAACCCTCC-3′; Anxa10 forward, 5′-CTCCTGCTTATGCGTTGGTT-3′, and reverse, 5′-GCTCAGAGCCTAATCAGCTTACC-3′.

### Statistics

All data are presented as the mean ± S.E.M. We analyzed and described the statistical significance of differences between groups according to an unpaired *t*-test and one-way or two-way analysis of variance followed by the Bonferroni multiple comparisons test. Data were carried out with GraphPad Prism 8.0 (GraphPad Software, La Jolla, CA, USA). A p value of < 0.05 was considered to reflect significance.

## Results

### Effects of incision and electrocautery on allodynia in mice

We first attempted to develop a new mouse model of persistent postsurgical pain by electrocautery treatment (Fig. 1A), and measured mechanical allodynia in the electrocautery, incision (Brennan’s model) and sham control groups by the von Frey test (Fig. 1B). While only transient allodynia was observed in incision-treated mice in response to mechanical stimuli, both persistent allodynia and transient allodynia were observed in response to mechanical stimuli in electrocautery-treated mice (Fig. 1C, Two-way repeated measures ANOVA with post-hoc Bonferroni test, **p < 0.01, ***p < 0.001 vs. Sham group, ^#^p < 0.05, ^##^p < 0.01, ^###^p < 0.001 vs. incision group).

### Changes in mRNA expression of Sprr1a and Anxa10 after electrocautery treatment

Our preliminary RNA-seq study to examine upregulated mRNA levels after electrocautery indicated that the levels of Sprr1a and Anxa10 in the spinal cord were potently increased after electrocautery (data not shown). To investigate the relationship between Sprr1a or Anxa10 and pain-processing in electrocautery-treated mice, we analyzed the expression of these genes by RT-qPCR in the ipsilateral side of the spinal cord. These experiments showed that the mRNA level of Sprr1a was significantly increased at 3 to 11 days after surgery in electrocautery-treated mice (Fig. 1D, One-way ANOVA with post-hoc Bonferroni test, **p < 0.01, ***p < 0.001 vs. Sham group, ^##^p < 0.01, ^###^p < 0.001 vs. incision group), whereas it was significantly increased at 3 days (Fig. 1D, One-way ANOVA with post-hoc Bonferroni test, **p < 0.01 vs. Sham group), but not at 7 or 11 days, after surgery in incision-treated mice. In addition, the mRNA level of Anxa10 was significantly and dramatically increased at 7 and 11 days (Fig. 1E, One-way ANOVA with post-hoc Bonferroni test, ***p < 0.001 vs. Sham group, ^###^p < 0.001 vs. incision group) after surgery in electrocautery-treated mice, whereas it was not significantly changed at 3 to 11 days after surgery in incision-treated mice.

### Analysis of chronic postsurgical pain-activated neurons

**Fig. 2:**
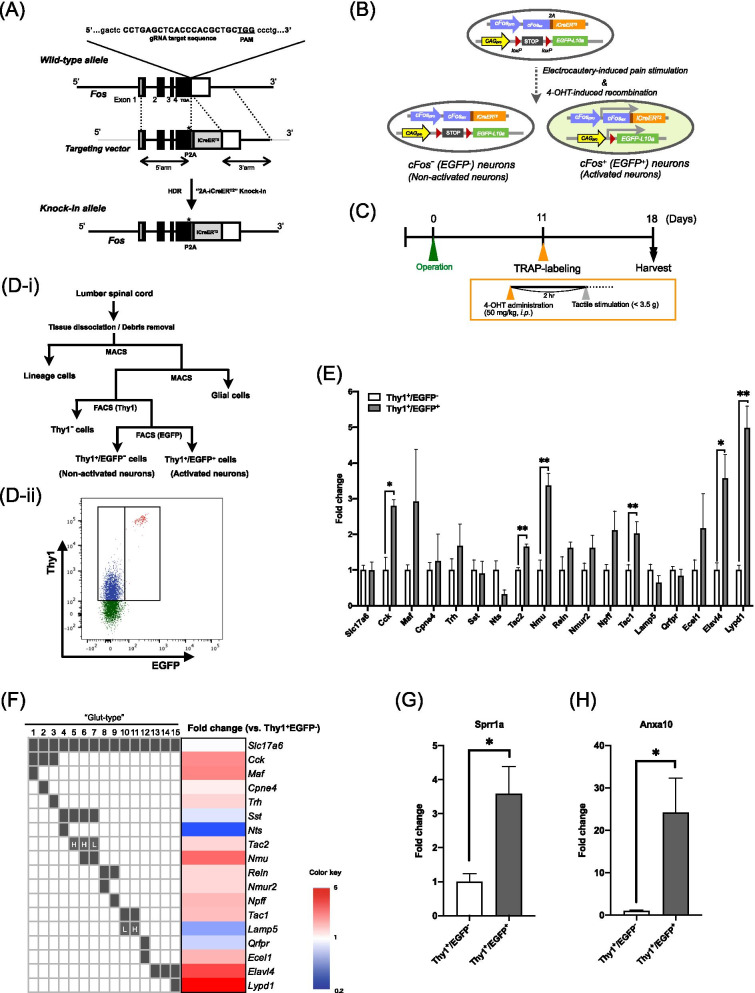
Analysis of chronic postsurgical pain-activated neurons. **A** Targeting strategy for the Fos-2A-iCreER^T2^ KI allele. PAM, protospacer adjacent motif. HDR, homology directed repair. Star; Two synonymous mutations (377L (CTG to TTA) and 378L (CTG to TTA)) were introduced to prevent binding and re-cutting of the sequence by gRNA after HDR. **B** Schematic diagram of EGFP labeling of electrocautery-activated neurons. iCreER^T2^ expression is driven by the activity-dependent cFos promoter to mediate 4-OHT-dependent recombination that permanently labels the active neurons with EGFP. (cFos-negative neurons: EGFP^−^, cFos-positive neurons: EGFP^+^) **C** Protocol for EGFP-labeling of electrocautery-activated neurons. Workflow for isolation of electrocautery-activated neurons from the ipsilateral side of the spinal cord by MACS and FACS. **D**-ii Representative FACS plot. Fluorescence from cells double-labeled for Thy1 (APC on Y axis) and cFos-EGFP (X axis) logarithmic plots (a log10 scale). In Thy1-labeled neurons, cFos-negative (EGFP^−^) and cFos-positive (EGFP^+^) neurons (left and right gates, respectively) on the X axis were represented by blue (left gate, cFos-negative (EGFP^−^)) and red (right gate, cFos-positive (EGFP^+^)) dots. **E** mRNA expression levels of markers of Slc17a6^+^ excitatory neurons in the spinal dorsal horn in electrocautery-activated neurons (cFos-positive neurons) compared to those in cFos-negative neurons. (n = 4–5 animals per sample, unpaired *t*-test, *p < 0.05, **p < 0.01 vs. cFos-negative neurons) **F** Heat map analysis of transcriptional profiles related to the subpopulation (“Glut 1-, 2-, 3-, 4-, 5-, 6-, 7-, 8-, 9-, 10-, 11-, 12-, 13-, 14- or 15-type”) of Slc17a6^+^ excitatory neurons in electrocautery-activated neurons. **G**, **H** Quantitative analysis of mRNA levels of Sprr1a **G** and Anxa10 **H** in cFos-positive neurons as compared to cFos-negative neurons. Each column represents the mean ± S.E.M. of 3 samples (n = 4–5 animals per sample, unpaired *t*-test, *p < 0.05 vs. cFos-negative neurons)

To investigate whether the increase in the expression of these genes is caused in neurons activated by electrocautery-induced pain signaling, we next analyzed chronic postsurgical pain-activated neurons by targeted recombination in active populations (TRAP)-labeling. To generate cFos-2AiCreER^T2^ mice that expressed P2A-iCreER^T2^ under the control of the immediate-early gene, cFos promoter, we inserted the P2A-iCreER^T2^ cassette at the mouse *Fos* locus [13] in C57BL/6 mice by CRISPR/Cas-mediated genome engineering (Fig. 2A). To label pain-activated cells, we next crossed cFos-2AiCreER^T2^ mice with LSL-EGFP/Rpl10a mice that expressed enhanced green fluorescent protein (EGFP) after Cre-mediated deletion of a loxP-flanked STOP cassette, and generated cFos-EGFP mice that specifically expressed EGFP in neurons, where iCreER^T2^ expression was induced by nerve firing in the presence of 4-OHT (Fig. 2B). Eleven days after electrocautery treatment of the hind paw of cFos-EGFP mice, these mice were injected with 4-OHT and pain-activated cells were labeled with EGFP (Fig. 2B, C). Seven days after 4-OHT injection, we performed FACS to isolate electrocautery-activated cFos-positive neurons (EGFP^+^) and cFos-negative neurons (EGFP^−^) in the ipsilateral side of the lumbar spinal cord of cFos-EGFP mice (Fig. 2D) followed by RT-qPCR to assess changes in gene expression by electrocautery. A recent single-cell sequencing study found that the excitatory neurons in the spinal dorsal horn can generally be divided into 15 subpopulations (“Glut” type 1 ~ 15) based on the similarity of their gene expression profiles [14]. After isolating electrocautery-activated cFos-positive neurons (EGFP^+^), we analyzed which types of neurons could be activated by the electrocautery. As a result, we revealed that the mRNA levels of cholecystokinin (Cck) identified as “Glut 1-, 2- and 3-types”, tachykinin 2 (Tac2) identified as “Glut 5- and 6-types”, neuromedin U (Nmu) identified as “Glut 6- and 7-types”, tachykinin 1 (Tac1) identified as “Glut 10-type”, and ELAV-like RNA binding protein 4 (Elavl4) and Ly6/Plaur domain containing 1 (Lypd1) identified as “Glut 15-type” in cFos-positive neurons were significantly higher than those in cFos-negative neurons (Fig. 2E, F, unpaired *t*-test, *p < 0.05, **p < 0.01 vs. cFos-negative neurons). The mRNA level of avian musculoaponeurotic fibrosarcoma oncogene homolog (Maf) identified as “Glut 1-type” in cFos-positive neurons was higher, albeit not significantly, than that in cFos-negative neurons (Fig. 2E, F). Among them, the expression of Elavl4 and Lypd1 was mostly and markedly predominant in cFos-positive neurons. Taken together with 15 subpopulations of spinal cord neurons based on gene expression profiles, these findings suggest that most of the electrocautery-activated cFos-positive neurons could be classified as “Glut 15-type” neurons. Under these conditions, the mRNA levels of Sprr1a and Anxa10 in cFos-positive neurons of electrocautery-treated mice at 11 days after surgery were significantly higher than those in cFos-negative neurons (Fig. 2G, H, unpaired *t*-test, *p < 0.05 vs. cFos-negative neurons).

### Long-lasting histone modifications of Sprr1a and Anxa10 genes after electrocautery treatment

**Fig. 3:**
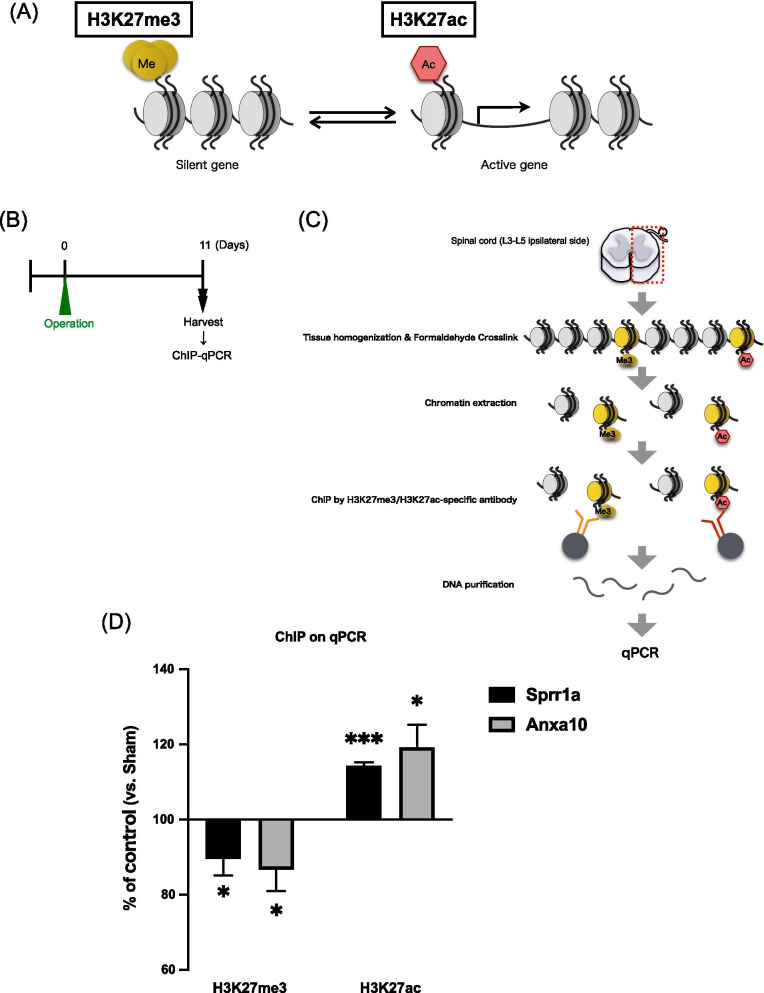
Long-lasting histone modification of Sprr1a and Anxa10 genes in the lumbar spinal cord after electrocautery treatment. **A** Schematic diagram of gene expression regulation by histone modification. **B** Workflow for experimental timeline. **C** Schematic diagram of ChIP-qPCR method. **D** Quantitative measurement of the levels of H3K27me3 (left) and H3K27ac (right) at the promoter region of the Sprr1a (black bar) and Anxa10 (gray bar) genes in the ipsilateral side of the lumber spinal cord at 11 days after electrocautery treatment. Each column represents the mean ± S.E.M. of 6 samples (H3K27me3, n = 5 animals per sample) or 3 samples (H3K27ac, n = 5 animals per sample) (unpaired *t*-test, *p < 0.05, ***p < 0.001 vs. sham group)

To investigate whether epigenetic modifications could be involved in the increased expression of these genes, we performed chromatin immunoprecipitation assays and quantified the amount of DNA modified with H3K27me3, which is known to suppress gene expression, or H3K27ac, which is known to promote gene expression in the ipsilateral side of the spinal cord collected at 11 days after the electrocautery operation (Fig. 3A–C). As a result, electrocautery-treatment significantly decreased the level of H3K27me3 at the promoter region of the Sprr1a and Anxa10 genes in the ipsilateral side of the spinal cord compared with that in sham-operated mice, while it produced a significant increase in the level of H3K27ac at the promoter region of the Sprr1a and Anxa10 genes in the ipsilateral side of the spinal cord (Fig. 3D, unpaired *t*-test, *p < 0.05, ***p < 0.001 vs Sham group).

### Effect of an H3K27 demethylase inhibitor on electrocautery-induced allodynia

**Fig. 4:**
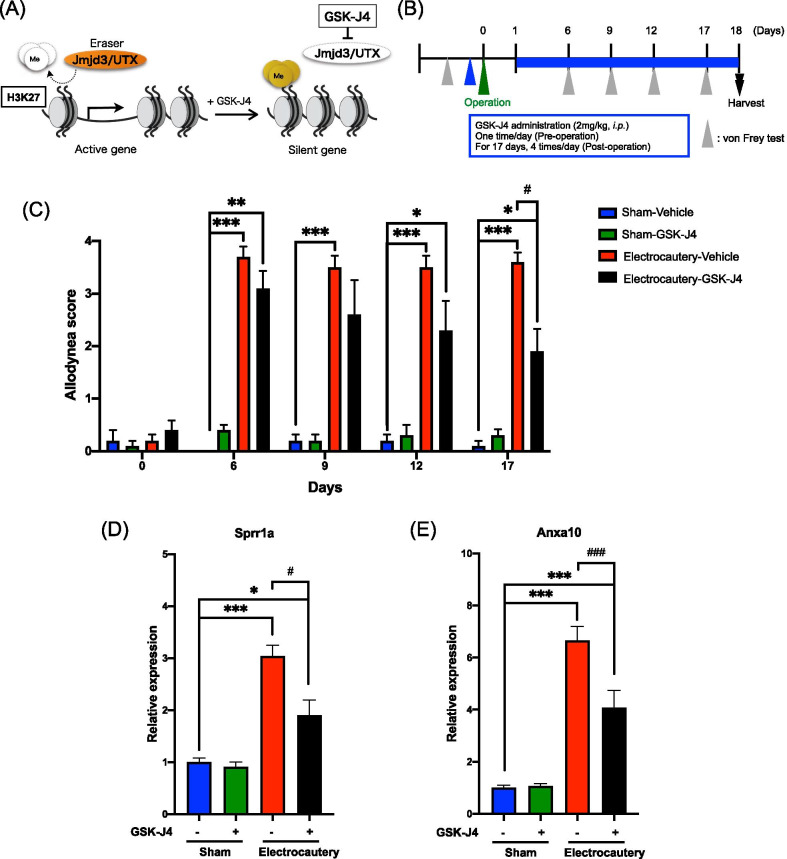
Effect of an H3K27 demethylase inhibitor on electrocautery-induced allodynia. **A** Schematic diagram of the mechanism of action of GSK-J4. **B** Protocol for measurement of the pain threshold and drug administration. **C** Changes in the pain threshold as measured by the von Frey test (0.16 g). Each point represents the mean ± S.E.M. of 5 mice (Two-way repeated measures ANOVA with post-hoc Bonferroni test, *p < 0.05, **p < 0.01, ***p < 0.001 vs. Sham-Vehicle group, ^#^p < 0.05 vs. Electrocautery-Vehicle group). **D**, **E** The mRNA levels of Sprr1a **D** or Anxa10 **E** in the ipsilateral side of the spinal cord were measured at 18 days after surgery. Each column represents the mean ± S.E.M. of 5 independent experiments (One-way ANOVA with post-hoc Bonferroni test, *p < 0.05, ***p < 0.001 vs. Sham-Vehicle group, ^#^p < 0.05, ^###^p < 0.001 vs. Electrocautery-Vehicle group)

Finally, to investigate whether such histone modifications could affect pain thresholds in electrocautery-treated mice, we evaluated the effect of GSK-J4 [15], a selective H3K27 demethylase inhibitor, on the electrocautery-induced tactile allodynia response following the von Frey test (Fig. 4A, B). Interestingly, persistent allodynia at 17 days after electrocautery was dramatically suppressed in GSK-J4-treated mice compared to that in vehicle-treated mice (Fig. 4C, Two-way repeated measures ANOVA with post-hoc Bonferroni test, *p < 0.05, **p < 0.01, ***p < 0.001 vs. Sham-Vehicle group, ^#^p < 0.05 vs. Electrocautery-Vehicle group). Consistent with our behavioral data, electrocautery-induced overexpression of Sprr1a and Anxa10 was significantly reduced by systemic administration of GSK-J4 (Fig. 4D, E, One-way ANOVA with post-hoc Bonferroni test, *p < 0.05, ***p < 0.001 vs. Sham-Vehicle group, ^#^p < 0.05, ^###^p < 0.001 vs. Electrocautery-Vehicle group).

## Discussion

Electrocautery can be used in most surgical procedures. Unlike with an incision created using a scalpel, the use of a heated electrode separates living (soft) tissues, including neurons, accompanied by hemostasis. Even if neurons are damaged by injury and/or incision, most acute pain during the healing period will respond to treatment with an analgesic medication [16]. On the other hand, the difficulty of recovery from tissue and neuronal damage caused by electrocautery can lead to chronic pain caused by changes in the nociception-related neuronal system [17]. In the present study, thermal allodynia produced after plantar incisions was observed with both a scalpel and electrocautery (data not shown), whereas prolonged mechanical allodynia was recognized only after electrocautery, indicating that electrocautery may influence the persistent hypersensitivity in response to mechanical stimuli after surgery.

The spinal cord is an important relay site for transmitting peripheral pain stimuli to the brain, and is an important target of medication to relieve pain. Therefore, we focused on gene expression in the spinal cord after incisions. It has been demonstrated that Sprr1a protein is only expressed in injured neurons and axons, and prompts axonal outgrowth [18]. Anxa10 is a member of the annexin family and plays an important role in various physiological processes such as cell differentiation and proliferation [19]. In recent studies, Anxa10 has been implicated in the development of neuropathic pain [20–22]. The mRNA level of Anxa10 has been shown to be persistently increased in the spinal cord after sciatic nerve ligation [20]. In the present study, we found that the mRNA levels of both Sprr1a and Anxa10 in the spinal cord were more potently and persistently increased by electrocautery than by incision. Taken together, these results suggest that increased levels of both Sprr1a and Anxa10 in the spinal cord may be, at least in part, associated with the establishment of CPSP after surgery.

Phosphorylation of phosphorylated-protein kinase C (PKC) located in the dorsal horn is critical for neuropathic pain [23–25]. Furthermore, PKCγ in interneurons transmits injury-induced mechanical allodynia [26]. In our preliminary study, immunoreactivity for phosphorylated pan-PKC (including PKCγ) in the spinal cord was dramatically enhanced by electrocautery (data not shown). Notably, there is some co-localization of PKCγ and Sprr1a in the spinal cord [27]. A growing body of evidence suggests that the activity of PKC is generally associated with the activity of extracellular regulatory kinase (ERK) in the spinal cord under a chronic pain-like state [28]. Spinal ERK signaling and subsequent release of tumor necrosis factor-α (TNF-α) and interleukin-1β has been reported to be a downstream pathway of Anxa10 [21]. The spinal Anxa10/NF-κB/MMP-9 pathway has also been shown to be involved in sciatic nerve ligation-induced neuropathic pain [22]. These findings suggest that electrocautery-activated neurons in the dorsal horn of the spinal cord may express Sprr1a and Anxa10.

To confirm whether Sprr1a and Anxa10 could play a critical role in long-lasting pain sensation after electrocautery, we next identified and characterized electrocautery-activated neurons in the spinal cord using FACS to isolate cFos-positive neurons in the ipsilateral side of the spinal cord of cFos-EGFP mice with electrocautery. As a result, the mRNA levels of Sprr1a and Anxa10 in cFos-positive neurons obtained from mice after electrocautery were much higher than those in cFos-negative neurons. It has been considered that the spinal dorsal horn neurons are a heterogeneous population. More recently, Häring et al. [14] identified 15 inhibitory and 15 excitatory molecular subtypes of spinal dorsal horn neurons and validated the existence of all of these identified neuron types in vivo. Thus, using the markers identified for each “subtype”, we found that most of the electrocautery-activated cFos-positive neurons could be classified as “Glut 15-type” neurons. “Glut 15-type” neurons, which highly express Lypd1, are located not only in superficial layers but also in deeper laminae. Moreover, “Glut 15-type” neurons have been shown to be activated by both noxious heat and cold stimuli, and account for the vast majority of spinobrachial projection neurons [14]. The parabrachial nucleus (PBN) is the most prominent locus of calcitonin gene-related peptide (CGRP)-expressing neurons in the brain and has been associated with the induction of threat memories and pain-related behaviors [29, 30]. Another study demonstrated that prolonged nociceptive signaling following nerve injury elicits the plasticity and sensitization of glutamatergic lateral PBN neurons, which leads to the development of persistent neuropathic pain [31]. These findings suggest that severe damage to tissues and neurons during electrocautery may contribute to CPSP through the activation of spinal neurons projecting to the parabrachial nucleus, which induces threat memories and the development of persistent pain, concurrent with the excessive production of core pain-related molecules, such as Sprr1a and Anxa10.

It has been widely accepted that epigenetic modulation persistently changes gene expression with no changes in the primary DNA sequence. A growing body of recent evidence suggests that epigenetic phenomena contribute to chronic pain as well as learning, memory, depression and drug addiction [32, 33]. Acetylation of most histone subunits, at any of several Lys residues, including Lys27 of histone 3 (H3), leads to the promotion of gene transcription, whereas histone methylation at Lys27 of H3 is well recognized to be strongly associated with gene repression. Here, we demonstrated that electrocautery significantly decreased the level of H3K27me3 with a significant increase in the level of H3K27ac at the promoter region of the Sprr1a and Anxa10 genes in the spinal cord. In addition, we found that treatment with GSK-J4, a selective H3K27 demethylase inhibitor, significantly suppressed electrocautery-induced persistent allodynia and overexpression of Sprr1a and Anxa10 mRNA. These findings suggest that the persistent production of core pain-related molecules, Sprr1a and Anxa10, with histone modifications in spinal cord neurons may be an essential mechanism underlying electrocautery-induced CPSP.

## Conclusion

We found for the first time that the concomitant expression of core pain-related molecules, Sprr1a and Anxa10, in the spinal cord was observed almost exclusively in electrocautery-activated neurons due to histone modifications. The present study suggests that neuronal damage associated with electrocautery leads to CPSP along with epigenetic modifications.

## Supplementary Information

**Additional file 1:Table S1. **Details of RT-qPCR primer.

## Data Availability

All of the data generated and analyzed in this study are included in this published article.

## Abbreviations

4-OHT: 4-Hydroxytamoxifen
Anxa10: Annexin A10
Cck: Cholecystokinin
ChIP: Chromatin immunoprecipitation
Cpne4: Copine IV
CPSP: Chronic postsurgical pain
Ecel1: Endothelin converting enzyme-like 1
EGFP: Enhanced green fluorescent protein
Elavl4: ELAV-like RNA binding protein 4
ERK: Extracellular regulatory kinase
FACS: Fluorescence-activated cell sorting
Gapdh: Glyceraldehyde 3-phosphate dehydrogenase
H3K27ac: Acetylated histone H3 at Lys27
H3K27me3: Tri-methylated histone H3 at Lys27
Lamp5: Lysosomal-associated membrane protein family, member 5
Lypd1: Ly6/Plaur domain containing 1
MACS: Magnetic-activated cell sorting
Maf: Avian musculoaponeurotic fibrosarcoma oncogene homolog
Nmu: Neuromedin U
Nmur2: Neuromedin U receptor 2
Npff: Neuropeptide FF-amide peptide precursor
Nts: Neurotensin
PBN: Parabrachial nucleus
PKC: Protein kinase C
Qrfpr: Pyroglutamylated RFamide peptide receptor
Reln: Reelin
Slc17a6: Solute carrier family 17 member 6
Sprr1a: Small proline rich protein 1A
Sst: Somatostatin
Tac1: Tachykinin 1
