# Supplementary material for: Histone modification of pain-related gene expression in spinal cord neurons under a persistent postsurgical pain-like state by electrocautery
Source: Mol Brain. 2021 Sep 20;14:146. doi: 10.1186/s13041-021-00854-y (PMC8451106; doi:10.1186/s13041-021-00854-y)
Supplement: Supplementary file 1 — Additional file 1:Table S1. Details of RT-qPCR primer. [file 13041_2021_854_MOESM1_ESM.pdf]

Table S1. Details of RT-qPCR primer

Gene symbol	Forward primer (5’→3’)	Reverse primer (5’→3’)
Anxa10	TGATGGATGCCCAAATGCTA	CCGGCCATACATGCTCTGAT
Cck	CTGCTAGCGCGATACATCCA	GTTCAAATGCGGGCAGGAAA
Cpne4	CCAGAAGCCCAAGGAATGAAG	GAGGATGACACAAGGGTCCG
Ecel1	CCTGCAGGTGCTGACAGACA	TGGGAGAGTCCTTGGGACAGT
Elavl4	GCCTCGATCAGGGATGCTAA	TGATGATGCGACCGTATTGAG
Gapdh	CATGGCCTTCCGTGTTCCTA	GATGCCTGCTTCACCACCTT
Lamp5	CACGAACCCCGAGAAAGACA	ATTGCTGGCCCACACATCAT
Lypd1	TCCAGCTGAACAACGATTGC	CGGTACATGATCCCAGCACTT
Maf	CCCCTGGCCATGGAATATGT	GGTCTCCACCGGTTCCTTTT
Nmu	GTCCTCTGTTGTGCATCCGT	CGTTGCGTGGCCTGAATAAA
Nmur2	GACCCAAGCGCAGTGACCTA	TCACCAGGCACACCAGAAGA
Npff	CCACCACAGTATGCCCACATT	AGGCTGGGCTCCTTCTAGGT
Nts	GTCTGTGCTCAGATTCAGAAGA	TTCCAAGACGGAGGACTTGC
Qrfpr	CGTCACGATGCTCCAGAACA	TGGTGAGGATTTCCGTCACA
Reln	CCATACTGTGGCCATGACTG	CACCTGGTTGTCCATGTGAG
Slc17a6	ACCCAGCCTGTCATGGGATA	GCTCCAGCATAGGAACCACAA
Sprr1a	ATGAGTTCCCACCAGCAGAAG	GCAGGGATCCTTGGTTTTGG
Sst	GACCCCAGACTCCGTCAGTT	TCATTCTCTGTCTGGTTGGGC
Tac1	AGGAAATCGATGCCAACGAT	GGGCGATTCTCTGCAGAAGA
Tac2	ATGCACGACTTCTTTGTGGGA	AAAGCTGGGGGTGTTCTCTTC
Trh	GGCCAGAACGTCGATTCTTG	GCTGGCGTTTTGTGATCCA

Table S1
